# Differences in expression rather than methylation at placenta-specific imprinted loci is associated with intrauterine growth restriction

**DOI:** 10.1186/s13148-019-0630-4

**Published:** 2019-02-26

**Authors:** Ana Monteagudo-Sánchez, Marta Sánchez-Delgado, Jose Ramon Hernandez Mora, Nuria Tubío Santamaría, Eduard Gratacós, Manel Esteller, Miguel López de Heredia, Virgina Nunes, Cecile Choux, Patricia Fauque, Guiomar Perez de Nanclares, Lauren Anton, Michal A. Elovitz, Isabel Iglesias-Platas, David Monk

**Affiliations:** 1 Imprinting and Cancer Group, Cancer Epigenetics and Biology Program, Bellvitge Biomedical Research Institute – IDIBELL, Av. Gran Via de L’Hospotalet 199-203, 08907 L’Hospitalet de Llobregat, Barcelona, Spain; 2 Fetal I+D Fetal Medicine Research Center, BCNatal - Barcelona Center for Maternal-Fetal and Neonatal Medicine, Hospital Clínic and Hospital Sant Joan de Déu, Barcelona, Spain; 3 Cancer Epigenetics group, Cancer Epigenetics and Biology Program, Bellvitge Biomedical Research Institute – IDIBELL, Gran via, L’Hospitalet de Llobregat, Barcelona, Spain; 4 Department of Physiological Sciences II, School of Medicine, University of Barcelona, Barcelona, Catalonia Spain; 5 Institucio Catalana de Recerca i Estudis Avançats, Barcelona, Catalonia Spain; 6 Human Molecular Genetics group, Genes, disease and Therapy Program, Bellvitge Biomedical Research Institute - IDIBELL, Av. Gran Via de L’Hospitalet 199-203, 08907 L’Hospitalet de Llobregat, Barcelona, Spain; 7 Centro de Investigaciòn Biomédica en Red de Enfermedades Raras (CIBERER), Madrid, Spain; 8 Université Bourgogne Franche-Comté - INSERM UMR1231, F-21000 Dijon, France; 9 (Epi) Genetics Laboratory, BioAraba National Health Institute, Hospital Universitario Araba-Txagorritxu, Vitoria-Gasteiz, Alava, Spain; 10 Maternal and Child Health Research Program, Department of Obstetrics and Gynecology, Center for Research on Reproduction and Women’s Health, University of Pennsylvania, Philadelphia, USA; 11 GReN (Grup de Reçerca en Neonatologia), BCNatal - Barcelona Center for Maternal-Fetal and Neonatal Medicine, Institut de Reçerca Sant Joan de Déu, Barcelona, Spain; 12 Leibniz Institute on Aging, Jena, Germany

**Keywords:** Imprinting, Placenta, DNA methylation, Epigenetics

## Abstract

**Background:**

Genome-wide studies have begun to link subtle variations in both allelic DNA methylation and parent-of-origin genetic effects with early development. Numerous reports have highlighted that the placenta plays a critical role in coordinating fetal growth, with many key functions regulated by genomic imprinting. With the recent description of wide-spread polymorphic placenta-specific imprinting, the molecular mechanisms leading to this curious polymorphic epigenetic phenomenon is unknown, as is their involvement in pregnancies complications.

**Results:**

Profiling of 35 ubiquitous and 112 placenta-specific imprinted differentially methylated regions (DMRs) using high-density methylation arrays and pyrosequencing revealed isolated aberrant methylation at ubiquitous DMRs as well as abundant hypomethylation at placenta-specific DMRs. Analysis of the underlying chromatin state revealed that the polymorphic nature is not only evident at the level of allelic methylation, but DMRs can also adopt an unusual epigenetic signature where the underlying histones are biallelically enrichment of H3K4 methylation, a modification normally mutually exclusive with DNA methylation. Quantitative expression analysis in placenta identified two genes, *GPR1-AS1* and *ZDBF2*, that were differentially expressed between IUGRs and control samples after adjusting for clinical factors, revealing coordinated deregulation at the chromosome 2q33 imprinted locus.

**Conclusions:**

DNA methylation is less stable at placenta-specific imprinted DMRs compared to ubiquitous DMRs and contributes to privileged state of the placenta epigenome. IUGR-associated expression differences were identified for several imprinted transcripts independent of allelic methylation. Further work is required to determine if these differences are the cause IUGR or reflect unique adaption by the placenta to developmental stresses.

**Electronic supplementary material:**

The online version of this article (10.1186/s13148-019-0630-4) contains supplementary material, which is available to authorized users.

## Background

In developed countries, intrauterine growth restriction (IUGR) is commonly associated with underlying placental insufficiency that prevents the fetus from achieving its full growth potential. Often confused with small for gestational age (SGA), IUGR is classified as aberrant growth in utero, whereas SGA refers to a newborn with a birth weight below the tenth centile for gestational age of the pregnancy [1]. The mechanisms leading to IUGR are not well defined and can be multifactorial. However, IUGR is clinically associated with increased umbilical or uterine artery resistance in prenatal Doppler measures, indicative of anomalies in placental vasculature. Abnormal remodelling of spiral arteries by invading trophoblast cells of the placenta is associated with pre-eclampsia (PE) [2]. Often pregnancies complicated by IUGR or PE are accompanied by prematurity, since early elective delivery is warranted to limit fetal distress [3]. In addition, IUGR babies not only have immediate medical problems requiring extensive stay in neonatal units but are at increased risk of common comorbidities later in life, including hypertension, type 2-diabetes, and heart disease through the effects of fetal programming [4].

The molecular mechanisms and genes involved in IUGR and PE are largely unknown. Changes in epigenetic modifications, including DNA methylation and histone modifications, have been suggested to play a role, contributing to stable alterations of gene expression. These epigenetic aberrations not only act as candidate biomarkers but may be maintained long after the initial developmental insult shaping metabolic phenotypes later in life [5].

In mammals, imprinted genes are known to regulate placental development, fetal growth and have recently been implicated in the aetiology of PE [6]. These genes are expressed in a parent-of-origin manner and are believed to have co-evolved with placentation [7]. Imprinted genes are associated with multiple layers of epigenetic regulation, including DNA methylation and histone tail modifications that results in their monoallelic expression [8]. Classically imprinted domains are regulated *in cis* by differentially methylated regions (DMRs) inheriting their methylation from the gametes that act as imprinting control regions (ICR). Several studies in humans have investigated changes in expression and regulation of imprinted genes in placentas from cohorts with heterogeneous clinical characteristics (healthy pregnancies, PE, SGA, and IUGR), suggesting that deregulation of this group of genes might play a role in regulating prenatal growth and development [9–12]. In addition, the epigenetic marks associated with imprinted genes have been shown to be particularly susceptible to variation following the use of assisted reproductive techniques [13], with initial studies in mice suggesting that methylation in the placenta is particularly vulnerable [14].

Recently several groups, including ours, have identified widespread placenta-specific imprinting which is restricted to humans [15–18]. Unlike conventional imprinting for which methylation at DMRs is normally ubiquitously observed in all tissues, placenta-specific imprinting is associated with transient germline DMRs inherited from the oocyte, present only in pre-implantation embryos and the placenta. This is in contrast to ubiquitous DMRs which can be either germline or somatically acquired. Furthermore, initial reports suggest that placenta-specific DMRs maybe polymorphic varying in frequency among individuals [16, 17].

We have carried out a comprehensive genome-wide profiling of bona fide ubiquitous and placenta-specific imprinted genes in a large collection of well-characterised placenta samples to address if aberrant methylation is associated with IUGR or PE. Furthermore, we looked for expression differences accounting for the IUGR. Lastly, we investigated the molecular mechanisms accounting for polymorphic imprinting observed at placenta-specific DMRs by comparing multiple epigenetic layers, revealing an involvement of post-translational histone modifications.

## Methods

### Samples

In a cohort of 127 placenta samples, 40 with corresponding maternal and cord blood samples were collected at the Hospital St. Joan De Deu (Barcelona, Spain), all of which had undergone microsatellite repeat analysis to confirm they were free of maternal DNA contamination (Additional file 1). For all samples, multiple sampling from the fetal side around the cord insertion site was taken, although for the majority of experiments only a single biopsy was used.

Chorionic villus sampling (CVS) from singleton gestations was obtained from Hospital Clinic Barcelona that subsequently resulted in normal live births at term. Following delivery, a term placenta biopsy was also collected. Both DNA and RNA extraction, as well as cDNA synthesis, were carried as previously described [19].

### Methylation array hybridization for 5mC analysis

We generated methylation datasets for 22 placenta samples using the Illumina Infinium Human Methylation450 BeadChip arrays, which simultaneously quantifies ~ 1.7% of all CpG dinucleotides. Two paired CVS-term placenta samples were hybridized to the Infinium MethylationEPIC (EPIC) arrays since the former platform was made obsolete in 2017, as were the samples from multiple anatomical sites and twin/triplet pregnancies. Bisulphite conversion was performed according to the manufacturer’s recommendations for the Illumina Infinium Assay (EZ DNA methylation kit, ZYMO, Orange, CA). The converted DNA was used for hybridisation following the Illumina Infinium HD methylation protocol at genomic facilities of the Cancer Epigenetics and Biology Program (Barcelona, Spain).

### Data filtering and analysis of methylation signals

In addition to the HM450k datasets generated in-house, we also included 45 placenta sample datasets (31 pre-eclampsia and 14 term controls), from GSE57767, which were collected at Hospital of the University of Pennsylvania (Additional file 1) [20]. Before analyzing the data, we excluded possible sources of technical biases that could influence results. We applied signal background subtraction, and inter-plate variation was normalised using default control probes in BeadStudio (version 2011.1_Infinium HD). We discarded probes with a detection *p* value > 0.01, containing single nucleotide polymorphisms (SNPs) within the interrogation or extension base as well as those with potential cross-reaction due to multiple sequence homologies. We also excluded probes that lacked signal values in one or more of the DNA samples analyzed. For the analysis of known imprinted domains, only probes mapping to DMRs with confirmed allelic methylation were examined. For samples hybridized to the EPIC, only probes present on the HM450k platform were analyzed. In-house bioinformatics R scripts were utilized for statistical comparisons.

### Statistical analyses

Differences between groups were checked by two-tailed unpaired Student’s *t* tests or Mann-Whitney-Wilcoxon tests depending on data distribution. These differences were considered significant at *p* < 0.05. The similarity between methylation profiles was measured by the Pearson correlation coefficients. For multiple comparisons, we used Benjamini and Hochberg corrections. All clinical data were introduced in a Statistical Package for Social Sciences (SPSS, IBM) software v17.0 database. Variables showing associations that were significant in the univariant analysis were subsequently introduced in multiple regression models to adjust for possible interactions or confounding factors. Results were considered significant if the *p* value < 0.05.

### Genotyping and imprinting analysis

Genotypes of potential SNPs were identified by interrogating the hg19 genome build on the UCSC sequence browser and confirmed by PCR and direct sequencing. Sequence traces were interrogated using Sequencher v4.6 (Gene Codes Corporation, MI) to distinguish heterozygous and homozygous samples. Heterozygous tissue samples were used for subsequent molecular technique that required allelic discrimination, including allelic RT-PCR, bisulphite PCR, and ChIP, with the SNP base incorporated into the PCR product (for primer sequences, see Additional file 2).

### RT-PCR analysis

#### Microfluidic-based quantitative expression analysis

Oligonucleotide primer sequences were designed using the Universal ProbeLibrary Assay Design Center on the Roche Resource webpage that also identified the appropriate UPL probe sequences for each amplicon (for primer sequences, see Additional file 2). First-strand cDNA for 50 placenta samples (a subset of those analyzed by pyrosequencing) was generated using supplier’s protocols for 50 ng of total RNA, which were subsequently pre-amplified (also referred to as specific target amplification; STA) for 14 cycles with a multiplexed pool of primers using the PreAmlification Master Mix (Fluidigm 100-5581). Subsequently, gene-specific RT-PCRs were performed with the same primers and specific UPLs on a Fluidigm 96.96 Dynamic Arrays using the Biomark HD system (Fluidigm Corp.) according to manufacturer’s instructions. The relative expression was quantified using the 2^−ΔΔCt^ method and represented as fold change in gene expression normalised to the mean of the housekeeping gene *RPL19* and the mean of all placentas. Only samples with two or more valid readings per triplicate were included.

#### Standard qRT-PCR

One microgram of RNA from 50 control samples from uncomplicated pregnancies was used to generate cDNA using random priming. These samples were used to determine the normal range of placental expression for *H19*, *IGF2*, *MEST*, and *SNU13* using SYBR Green mixture (SYBR® Green) on a 7900HT Fast Real-Time PCR (Applied Biosystems) instrument.

### Bisulphite methylation analyses

Allelic PCR: For standard bisulphite conversion, we used the EZ DNA Methylation-Gold kit (Zymo) following the manufacturer’s instructions. Approximately 2 μl of bisulphite-converted DNA was used in each amplification reaction using Immolase Taq polymerase (Bioline) for 45 cycles and the resulting PCR product sub-cloned into a pGEM-T easy vector (Promega) for sequencing (for primer sequences see Additional file 2).

Pyrosequencing: Standard bisulphite PCR was used to determine the methylation in a confirmatory cohort (see Additional file 1 for details). Standard amplification of 50 ng of bisulphite-converted DNA was used with the exception that one primer was biotinylated (for primer sequence see Additional file 2). The entire biotinylated PCR product (diluted to 40 μl) was mixed with 38 μl of binding buffer and 2 μl (10 mg/ml) streptavidin-coated polystyrene beads. After incubation at 65 °C, DNA was denaturated with 50 μl 0.5 M NaOH. The single-stranded DNA was hybridized to 40-pmol sequencing primers dissolved in 11 μl annealing buffer at 90 °C. For sequencing, a primer was designed to the opposite strand to the biotinylated primer used in the PCR reaction. The pyrosequencing reaction was carried out on a PyroMark Q96 instrument. The peak heights were determined using Pyro Q-CpG1.0.9 software (Biotage).

### Chromatin immunoprecipitations (ChIP)

ChIP on native chromatin was performed as previously described [21]. Essentially, ~ 100 mg of placenta tissue was disrupted to release nuclei using 0.5 mM zirconium beads (Sigma-Aldrich) in a Precellys 24 tissue homogenizer (Berton_Corp) which were subsequently isolated by centrifugation. The nuclei were subject to MNase digestion (15 units/ul who) for between 1 and 3 min and aliquots run on agarose gels to select digest with the most abundant mono- and pento-nucleosome fractions. Approximately 4 μg of chromatin was used for an immunoprecipitation reaction with protein A agarose/salmon sperm DNA (Thermo Fisher) and a H3K4me3 (Diagenode, C15410003-50), H3K4me2 (Millipore, 07-030), and H3K9me3 (Abcam, AB8898) antibodies. Each ChIP was performed alongside a mock immunoprecipitation with an unrelated IgG antiserum (12–371, Millipore), and a 50% fraction of the input chromatin was extracted in parallel. Before use in downstream experiments, control allelic precipitation at ubiquitous DMRs (*MEST* and *SNURF*) confirmed the quality of the ChIP material.

For quantitative analysis, the input and antibody-bound fractions were subject to real-time PCR amplification with a SYBR Green mixture (SYBR® Green) using a Quant Studio 5 or 7900HT Fast Real-Time PCR (Applied Biosystems) instrument. Background precipitation levels were determined from the mock precipitations with a non- specific IgG antiserum. Bound/input ratios were calculated and were normalised against the precipitation level at the *GAPDH* promoter for active marks and SAT2 repeats for H3K9me2/3. All PCRs reactions were performed three times in triplicate. The primers used are listed in Additional file 2.

## Results

### Aberrant methylation at ubiquitous DMRs is associated with IUGR

**Fig. 1:**
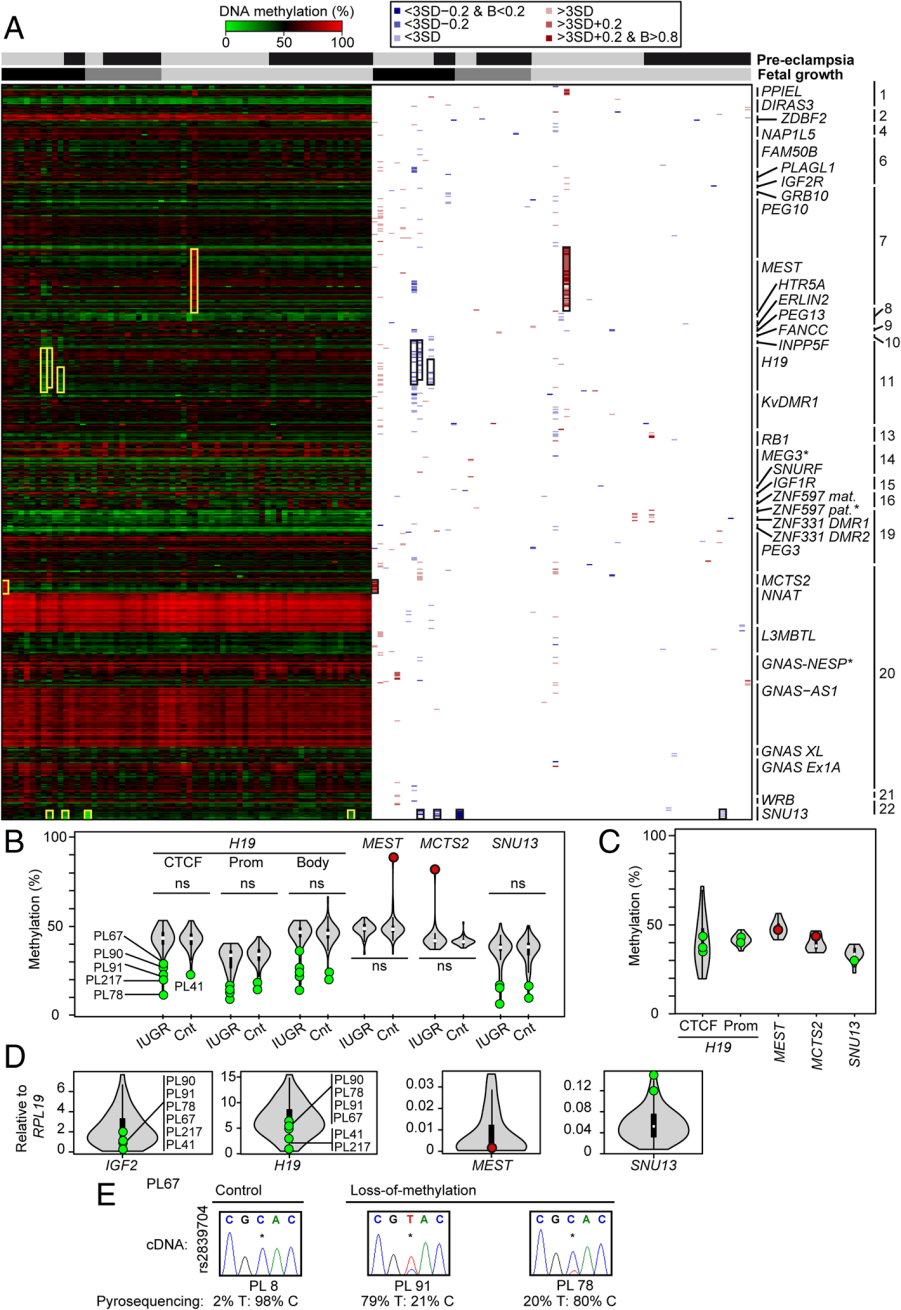
Analysis of imprinted methylation at ubiquitous DMRs using HM450k methylation arrays. **a** The left side is a heatmap of absolute methylation (*β* values) for individual Infinium probes mapping to 35 DMRs in 67 placenta samples. Samples with abnormal methylation across a DMR are highlighted by yellow boxes. The right side of the figure reveals methylation difference according to severity, with blue and red representing hypo- and hypermethylation, respectively. Samples are classified by both the presence of pre-eclampsia (black yes; gray no) and fetal growth parameters (appropriate for gestational age light gray; SGA dark gray; IUGR black). *Adjacent to the DMR name indicates somatic establishment of methylation. **b** Pyrosequencing confirmation of the aberrant methylation profiles identified using HM450k arrays, as well as the quantification of additional placenta samples. The violin plots used include the median (white dot) and the interquartile range (black rectangle), with hypomethylated samples identified using the HM450k array platform highlighted as green data points, while hypermethylated samples are in red. The non-parametric Mann-Whitney-Wilcoxon test was used to calculate the statistical significance of the differences between IUGR and control groups (ns indicated no significance, *p* > 0.05). **c** Pyrosequencing quantification in cord blood samples. The violin plots show the distribution of methylation for 16 controls. Samples with aberrant placenta methylation profiles are highlighted. **d** Quantitative RT-PCR for transcripts regulated by affected DMRs. The violin plots represent the expression levels of 50 control placenta samples with normal birthweight and methylation. Samples with hypomethylation are highlighted as green data points, while hypermethylated samples are in red. **e** Allelic expression analysis was performed for the *H19* transcript using the rs2839704 SNP, with allelic contributions quantified by pyrosequencing

Using the Illumina Infinium Human Methylation450 BeadChip arrays (HM450k) array platform, we initially profiled ubiquitous imprinted DMRs in 67 placenta samples. A total of 601 probes located within 35 ubiquitous DMRs passed quality control and were assessed in all samples. Overall, the methylation at these regions was extremely stable in control placentas and those from complicated pregnancies with no differences detected between groups. We identified isolated gain-of-methylation (GOM) at two DMRs associated with the *MEST* and *MCTS2* genes in a control biopsy (PL35) and an IUGR placenta (PL37), respectively. In addition, we observed multiple cases with partial loss-of-methylation (LOM) at the *H19* and *SNU13* DMRs. The three samples with hypomethylation of the *H19* ICR (PL67, 90, 217) were all from pregnancies complicated by IUGR, whereas the four samples with aberrant *SNU13* methylation were from IUGR (PL90), SGA (PL4002), and pre-eclampsia (PL2048, 2026)(Fig. 1a; Additional file 3).

Subsequently, we tested a second cohort of samples by quantitative pyrosequencing (*n* = 127, 109 not analyzed on HM450k platform) and confirmed the array observations (Fig. 1b). Interestingly, additional cases of *H19* ICR (PL78 and 41) and *SNU13* (PL78, 146) hypomethylation were detected in this extended sample set. This also revealed that there were no differences in methylation for any DMR when comparing the IUGR, SGA, and pre-eclampsia groups with controls and that previously identified hypomethylated samples were all in the lower quartile. To ensure that the isolated methylation anomalies were epigenetic in origin and not associated with chromosomal aberrations, the presence of micro-deletions or uniparental disomy were discounted using both standard polymorphic short tandem repeats and multiplex ligation-dependent probe amplification analyses (Additional file 4).

We have previously shown that the *H19* gene has a unique methylation profile in placenta compared to somatic tissues with the paternal allele-specific methylation extending from the core ICR and throughout the gene body [22]. To determine if the LOM we observed at the *H19* ICR is restricted to this interval, or is more widespread, we performed pyrosequencing for the promoter and gene body. This revealed that the partial hypomethylation was generally uniform throughout the interval (Fig. 1b). Hypomethylation of the paternal allele of the *H19* ICR is associated with the pre- and postnatal growth disorder Silver-Russell syndrome (SRS; OMIM 180860) [23]. Interestingly, all aberrant methylation profiles for the four DMRs were restricted to the placenta as corresponding cord blood samples were in the normal range, suggesting that the patients are not classic imprinting disorders (Fig. 1c).

Hypomethylation of *H19* results in biallelic expression of the *H19* non-coding RNA and the concomitant decrease of expression of *IGF2*. Using RT-PCR and Sanger sequencing in samples heterozygous for a SNP in exon 5, we observe LOM at *H19* results in the reactivation of the normally repressed paternal allele (Fig. 1d, e), accompanied by lower expression of *IGF2* compared to 50 control placenta samples (hypomethylated mean 0.51 ± 0.38 SD vs control mean 2.49 ± 1.6 SD, *p* < 0.01). Furthermore, the samples with *SNU13* LOM were among the samples with most abundant expression and the single placenta sample with *MEST* GOM was the lowest expressed (Fig. 1d). Due to the small number of samples with these isolated methylation aberrations, we could not perform additional statistical comparisons. Allelic studies could not be performed for the *SNU13* LOM samples due to the lack of heterozygosity for exonic SNPs.

### Polymorphic events at placenta-specific DMRs are not more frequent in pregnancy complications

**Fig. 2:**
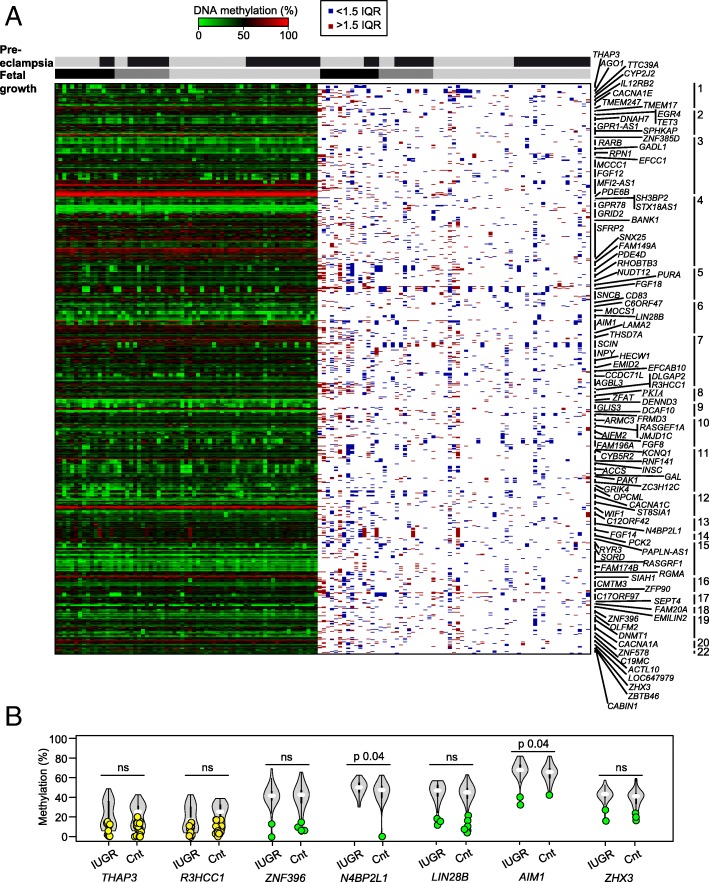
Analysis of imprinted methylation at placenta-specific DMRs using HM450k methylation arrays. **a** The left side is a heatmap of absolute methylation (*β* values) for individual Infinium probes mapping to 112 DMRs in 67 placenta samples. The right side of the figure reveals outlier samples as identified by > 1.5 IQR (red) and < 1.5 IQR (blue) methylation values, respectively. Samples are classified by both the presence of pre-eclampsia (black yes; gray no) and fetal growth parameters (appropriate for gestational age light gray; SGA dark gray; IUGR black) **b** Pyrosequencing confirmation of the aberrant methylation profiles identified using HM450k arrays, as well as the quantification of the extended placenta cohort. The violin plots include the median (white dot) and the interquartile range (black rectangle); samples with hypomethylation defined by < 1.5 IQR are highlighted as green data points. Placenta-specific DMRs with a distinct population of lowly methylated samples not identified by IQR statistic are highlighted in yellow. The non-parametric Mann-Whitney-Wilcoxon test was used to calculate the significance of the differences between IUGR and control groups (ns indicated no significance, *p* > 0.05)

We utilized the same Illumina Infinium HM450K array data to explore the profiles of placenta-specific DMRs. In total, we analyzed 112 regions incorporating 763 probes, which represents the total number of confirmed placenta-specific DMRs from our previous work [15, 16] and those reported in two additional studies [17, 18]. Consistent with previous reports, this revealed that the majority of placenta-specific DMRs have some samples with low-level methylation, indicative of a polymorphic event (Fig. 2a; Additional file 3).

To confirm these observations, 32 placenta-specific DMRs associated with paternal expression were analyzed by pyrosequencing. For two loci, *AIM1* and *N4BP2L1*, we found significant differences between the mean methylation levels for the IUGR group compared to controls (IUGR mean 66.5% vs control mean 63.0%, *p* 0.038; IUGR mean 52.5% vs control mean 48.6%, *p* 0.046, respectively). No significant differences were observed between pre-eclampsia samples and controls (Fig. 2b, Additional file 5). After separating groups by gender, two genes *AIM1* and *RHOBTB3* showed a statistically significant (*p* < 0.05), but biologically very small difference in percentage methylation, with higher levels observed in IUGR females. However, these differences did not withstand Benjamini and Hochberg correction for multiple testing (Additional file 5). In addition to comparing the mean methylation for each group, we also determined the number of placenta samples lacking methylation at each placenta-specific DMR. Using the ± 1.5 interquartile range (1.5 IQR) to determine outliers, we observed no differences in the frequency of these polymorphic events, with equal numbers of hypomethylated samples distributed between the IUGR and control groups (Fig. 2; Additional file 5). For some regions, such as *THAP3* and *R3HCC1*, there were two distinct populations one with low-level methylation (52% and 53% of the samples, respectively), not detected as outliers, and those maintaining partial methylation indicative of a DMR (Fig. 2; Additional file 6).

### Placenta-specific DMRs maintain methylation across gestation

One possible mechanism leading to the apparent polymorphic placenta-specific DMRs is the failure to maintain allelic methylation during gestation. For a temporal comparison, we performed methylation profiling using the EPIC methylation arrays on first trimester CVS and compared with corresponding samples at term. This revealed that DNA methylation level at placenta-specific DMRs is stable between the two points with higher correlations at placenta-specific DMRs (mean Pearson’s *r* = 0.88; range 0.85–0.90) than between unrelated placenta samples (mean Pearson’s *r* = 0.76; range 0.64–0.89) (Additional file 7). Detailed scrutiny of the paired samples revealed that the DMRs showing polymorphic LOM were maintained from first trimester to term but diverged between individuals (Additional file 7). In addition, to ensure that the methylation profiles observed were uniform across the placental plate, we determined the placenta-specific DMR profiles from multiple biopsies from the same term placentas. Biopsies collected from the opposite sides of the cord insertion site also showed higher correlations (mean Pearson’s *r* = 0.95; range 0.95–0.96) than between unrelated placenta samples suggesting that methylation does not vary greatly between sampling sites. Finally, we compared placenta samples from dizygotic twins and triplets. This revealed that the correlations between samples of the same gestations, therefore sharing the same in utero environment and maternal exposures, were also more similar (mean Pearson’s *r* 0.83; range 0.82–0.85) than between unrelated samples.

### Altered allelic histone modification profile is associated with biallelic expression

During our initial description of placenta-specific imprinting, we observed that polymorphic biallelic expression was associated with two different scenarios. The first one was the lack of methylation at the DMR resulting in the absence of imprinting. The second more complicated and rare event was associated with maintained maternal methylation [16]. This suggests that a subset of these DMRs do not influence allelic expression or that the expression arises from an alternative promoter. Since our previous searches failed to identify transcripts originating from upstream promoters in placenta, we favor the first option.

To understand the role of additional epigenetic mechanisms in the polymorphic placenta-specific imprinting, we studied post-translational modifications of histones using ChIP. After extensive allelic methylation and expression analyses of samples in our cohort, two regions, *LIN28B* and *R3HCC1*, were selected for in-depth ChIP analysis.

**Fig. 3:**
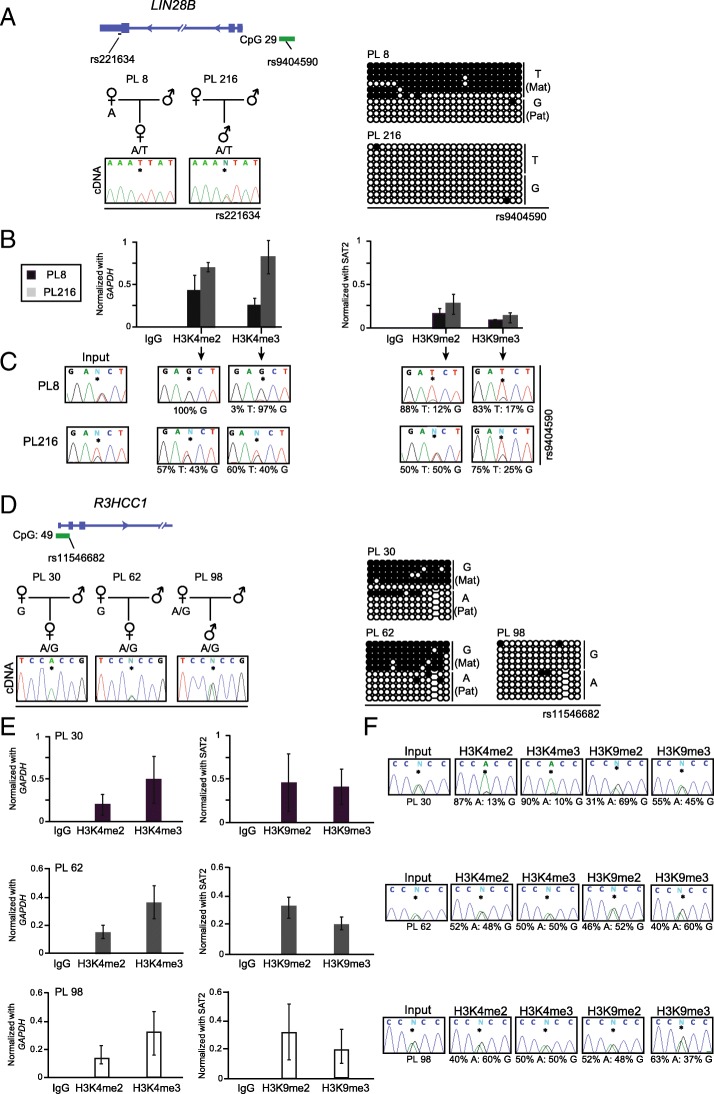
Detailed characterization of histone modifications within placenta-specific DMRs in samples with polymorphic imprinting. Schematic representation of **a** the *LIN28B* loci, indicating the position of transcripts and CpG islands incorporating the DMRs. The methylation of the two placenta samples analyzed was assessed by bisulphite PCR and sub-cloning. Each circle represents a single CpG on a DNA strand. (•) methylated cytosine, (o) unmethylated cytosine. Each row corresponds to an individual cloned sequence with the genotype indicated for the heterozygous SNP incorporated into the amplicon. **b** Quantitative PCR targeting the *LIN28B* DMR in ChIP material. Precipitations were normalised to *GAPDH* promoter (H3K4me2/3) and SAT2 repeats (H3K9me2/3). The graphs represent the mean values (± standard deviation). For each placenta sample, values are the mean of at least three independent ChIP experiments, each in triplicate. **c** The allelic distribution of each mark was determined by direct sequencing of the PCR product encompassing the heterozygous SNP used for the methylation analysis. The percentage of allelic enrichment is shown under the electropherograms. **d** Diagram of the *R3HCC1* loci and the methylation bisulphite PCR profiles of the three analyzed samples. **e**, **f** The quantitative and allelic ChIP results for the *R3HCC1* DMR, respectively, which were analyzed in the same way as for *LIN28B*

The maternally methylated region upstream of the *LIN28B* gene presents with lack of methylation in ~ 12% of samples. Interestingly, one of the samples lacking methylation, PL216, comes from a multiple gestation pregnancy with the dizygotic twin sibling (PL215) having a normal methylation profile at this locus, indicating that the maternal environment is not responsible for these differences (Additional file 8). For samples with maintained *LIN28B* imprinting (Fig. 3a), qPCR on ChIP material revealed enrichment for both active (H3K4me2 and H3K4me3) and repressive histone modifications (H3K9me2 and H3K9me3), which was shown to be on opposing chromosomes using allelic PCR (Fig. 3b, c). However, in the sample that was biallelically unmethylated, we observed increased enrichment of active histone marks on both parental chromosomes, while the amount of precipitation of repressive marks was largely unchanged.

In the case of the *R3HCC1* DMR, we identified samples with the three situations associated with polymorphic imprinted expression. One placenta with monoallelic expression and maternal methylation (PL30) and two others with biallelic expression, the first one of them with the expected ~ 50% methylation at the DMR (PL62) and the other lacking allelic methylation (PL98) (Fig. 3d). As expected, qPCR on ChIP material from control placenta samples with maternal methylation and monoallelic expression revealed opposing enrichment for the active (H3K4me2 and H3K4me3) and repressive histone (H3K9me2) modifications. However, in the samples with biallelic expression, active marks were enriched on both parental chromosomes, irrespective of allelic methylation status (Fig. 3e, f).

### Expression levels of imprinted genes are associated with IUGR

**Fig. 4:**
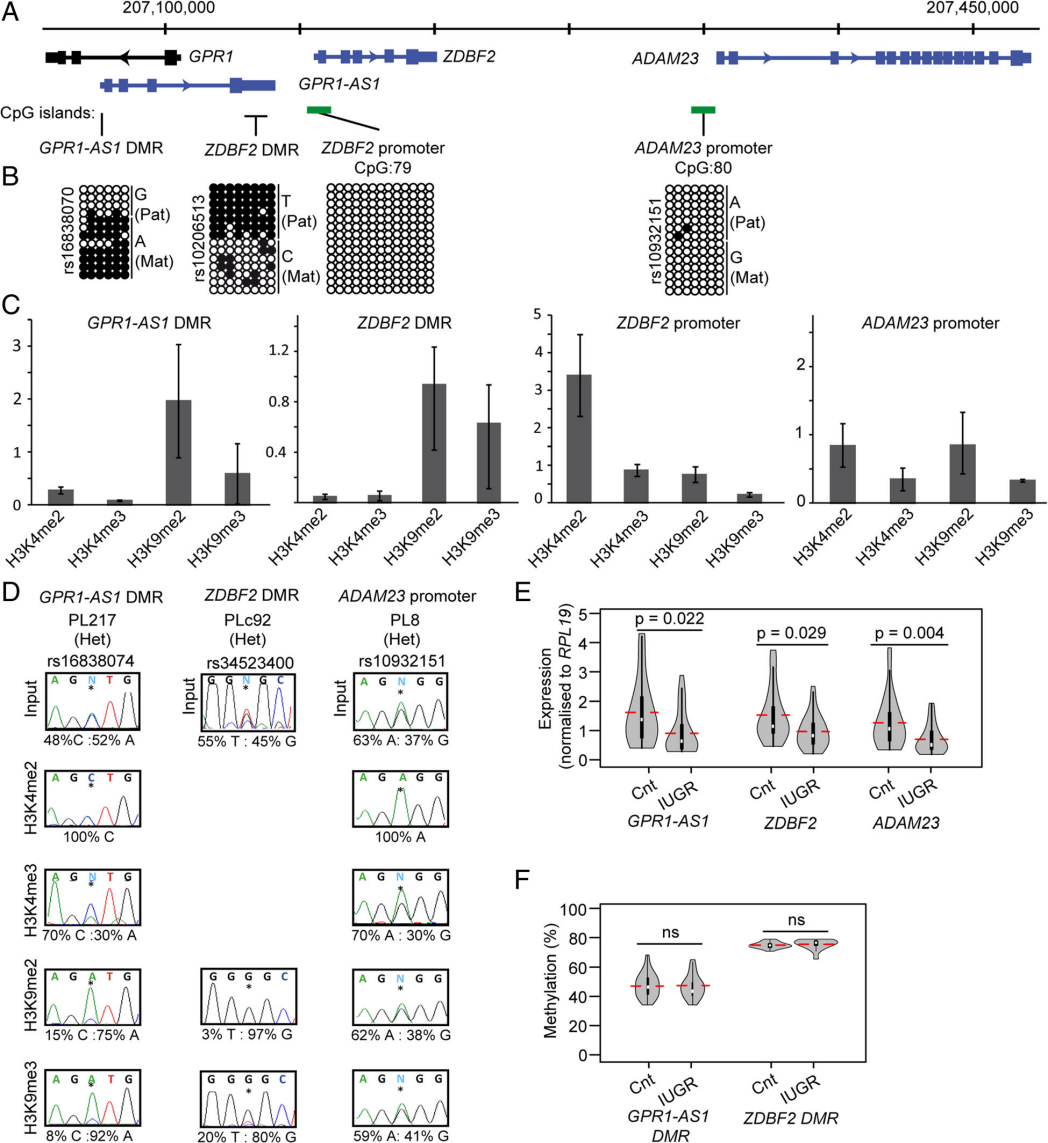
Epigenetic and transcriptional description of the *GPR1-AS1-ADAM23* locus in IUGR placentas. **a** Schematic representation of the *GPR1*-*AS1-ADAM23* imprinted locus on chromosome 2, indicating the position of the transcripts and CpG islands incorporating the DMRs. **b** Characterization of the DMRs and promoter CpG islands in placenta biopsies. Each circle represents a single CpG on a DNA strand. (•) methylated cytosine, (o) unmethylated cytosine. Each row corresponds to an individual cloned sequence with the genotype indicated for heterozygous SNP incorporated into the amplicon. **c** Quantitative PCR on ChIP material. Precipitations were normalized to *GAPDH* promoter (H3K4me2/3) and SAT2 repeats (H3K9me2/3). The graphs represent the mean values (± standard deviation). For each placenta sample, values are the mean of at least three independent ChIP experiments, each in triplicate. **d** The allelic distribution of each mark was determined by direct sequencing of the PCR product encompassing heterozygous SNPs. **e** Quantification of expression levels of *GPR1-AS1*, *ZDBF2*, and *ADAM23* using microfluidic-based RT-qPCR. The results are presented as violin plots, with the median (white dot), mean (red line), and the interquartile range (black rectangle) shown. All expression levels were normalised to the mean of the *RPL19* housekeeping gene. To determine the statistical significance of the difference between the IUGR and control groups, Student’s two-tailed *t* test was used and *p* values are indicated over the horizontal comparison lines. **f** Pyrosequencing quantification of the *GPR1*-AS1 and *ZDBF2* DMRs. The violin plots include the median (white dot) mean (red line) and the interquartile range (black rectangle). The non-parametric Mann-Whitney-Wilcoxon test was used to calculate the statistical significance of the differences between IUGR and control groups (ns indicated no significance, *p* > 0.05)

We have previously shown that significant differences in expression of transcripts are often independent of imprinted DMR methylation [24, 25]. To determine if absolute expression levels are associated with IUGR, we performed microfluidic-based quantitative RT-PCR analysis for genes associated with 22 ubiquitous and 13 placenta-specific DMRs. Unfortunately, we did not have sufficient samples with pre-eclampsia to perform statistical analysis. To identify genes significantly differentially expressed between IUGR and control samples, we carried out unpaired two-tailed *t* tests (*p* < 0.05). This revealed that only three ubiquitously imprinted genes were significantly differentially expressed; *ZNF331* was higher in IUGRs compared to controls (IUGR mean 1.32 vs control mean 0.78, *p* = 0.041), whereas *PEG10* (IUGR mean 1.05 vs control mean 1.57, *p* = 0.047) and *ZDBF2* (IUGR mean 0.98 vs control mean 1.5, *p* = 0.029) were significantly lower (Fig. 4e; Additional file 9). Four placenta-specific imprinted transcripts were also differentially expressed; *ADAM23* (IUGR mean 0.62 vs control mean 1.25, *p* = 0.004), *GPR1-AS1* (IUGR mean 0.87 vs control mean 1.55, *p* = 0.022), *LIN28B* (IUGR mean 1.01 vs control mean 1.36, *p* = 0.046), and *ZHX3* (IUGR mean 0.56 vs control mean 1.16, *p* = 0.004) all being less abundant in the IUGR population (Additional file 9).

Differences in IUGR-associated expression can be influenced by fetal gender [24]. We analyzed expression after separating the groups according to sex. Surprisingly, we observe that for five of the seven genes with expression differences (*PEG10*, *ZDBF2*, *ADAM23*, *LIN28B*, and *ZHX3*), the effect was primarily due to lower transcript abundance in the IUGR male group (Additional file 9). Furthermore, after separating by gender, we noticed a difference not identified in the population as a whole, with *DNMT1* (IUGR mean for males 0.62 vs control mean for males 1.42, *p* = 0.004) and *INPPF5* (IUGR mean for the placenta of males 0.87 vs mean control males 1.42, *p* = 0.018) being less abundant in IUGR males compared to controls, while *GRB10*, a potent growth inhibitor, is higher in IUGR males (IUGR mean for males 0.98 vs control mean for males 0.62, *p* = 0.026) (Additional file 9).

Clinical factors that were different between the IUGR and control groups as well as those that seem to be associated to the expression of each gene were explored (Additional file 10). Once the confounders (gestational age and birth weight, fetal gender, ART, and maternal characteristics) were controlled for by linear regression, only changes in *ZDBF2* and *GPR1-AS1* were associated with IUGR. For *LIN28B* and *ZHX3*, the results of the univariate analysis seemed to be explained by a confounding effect of gestational age.

### Downregulation of the *GPR1-AS1-ADAM23* imprinted domain in IUGR

Three of the genes showing altered expression in our IUGR population, *GPR1-AS1*, *ZDBF2*, and *ADAM23*, are located adjacent to one another within the chromosome 2q33 imprinted cluster (Fig. 4a). This domain is highly conserved between mammals. Studies in mice show that imprinting is dependent on a maternal germline placenta-specific *Gpr1-as* DMR, which subsequently influences the expression of the long isoform of *Zdbf2* (*Liz*), orthologous to *GPR1-AS1* in humans [26]. The *Liz* transcript is responsible for the *in cis* regulation of the paternally methylated DMR upstream of the canonical *Zdbf2* promoter during embryogenesis [27]. The observed downregulation we note in our placenta cohort suggests that there could be a synchronised deregulation throughout this domain in IUGR, which is supported by the moderate correlation between the expression of *GPR1-AS1* and *ZDBF2* (Pearson’s *r* = 0.38, *p* 0.001). To further investigate the possible mechanisms involved in this coordinated regulation, we confirmed the allelic methylation of the two DMRs, as well as characterising the epigenetic profile for the promoter CpG islands associated with *ZDBF2* and *ADAM23*. This revealed that in placenta, the promoters of *ZDBF2* and *ADAM23* are devoid of methylation, *GPR1-AS1* is maternally methylated as previously reported (26), and *ZDBF2* is preferentially methylated on the paternal allele (Fig. 4b). Furthermore, interrogation of the histone modifications located in these intervals revealed allelic enrichment of permissive marks on the expressed allele at the *ADAM23* promoter and *GRP1-AS1* DMR, with the later also decorated with H3K9me3 on the maternal allele. Only allelic H3K9 methylation was observed within the *ZDBF2* DMR (Fig. 4d, e). Unfortunately, we could not perform allelic ChIP within the *ZDBF2* promoter because of the lack of informative SNPs. Finally, we performed pyrosequencing to quantify the methylation at the two DMRs. No differences were observed between the means of the IUGR and control groups (Fig. 4f), with all samples being appropriately methylated, indicating that the variance in expression levels is independent of allelic methylation.

## Discussion

Abnormal placentation is responsible for numerous pregnancy complications including infertility, miscarriage, PE, and IUGR. Pre-eclampsia may manifest alongside IUGR [28] and both normotensive IUGR and PE are associated with a deficient invasion of specific extra-villous trophoblast. This results in the limited transfer of nutrients and exchange of waste products between the two genetically distinct individuals, fetus and mother, which have conflicting needs. Studies of imprinted genes in mouse models have revealed that they regulate growth in a manner that balances the supply and demand of maternal nutrients [29], with some paternally expressed genes driving fetal growth and maternally expressed genes restricting it via limiting resource allocations. Whilst not all imprinted genes support this hypothesis, it is endorsed by reports of increased expression of *CDKN1C*, a maternally expressed growth repressor [30], as well as lower paternal *IGF2* in SGA and IUGR placenta samples [9–11, 25, 31, 32]. In some cases, the genes have been proposed to influence fetal growth early in development, with significant growth correlations only observed in first trimester samples and not term, while others, including *PHLDA2*, are classified as late growth effectors with the largest impact observed at term [11].

For the majority of studies, to date, very few have assessed both methylation and expression in a genome-wide manner to determine the role of imprinting on pregnancy outcome (reviewed in [8]). Here, we describe the first large-scale assessment of methylation and expression at both ubiquitous and placenta-specific imprinted domains. This revealed that although rare, isolated LOM at ubiquitous DMRs can occur and does directly influence expression. We observe hypomethylation of *H19* in placenta, but not cord blood, associated with low levels of *IGF2* and concomitant *H19* biallelic expression in four IUGR cases. Such epimutations are normally associated with SRS, for which there is comparable hypomethylation between blood-derived DNA and placentas [33]. This isolated placenta *H19* hypomethylation is likely reflecting non-syndromic IUGR and not SRS. Consistent with this hypothesis, the individuals attain a low score using the Netchine-Harbison clinical criteria [34, 35] and exhibit post-natal catch-up growth once the constraints of the placenta were removed at delivery. It is currently unclear if the other epigenetic events are also involved in fetal growth, as hypomethylation of the *MCTS2P* DMR has been observed at a low frequency in large control cohort studies [36], but both *H19* hypomethylation and *MEST* hypermethylation have previously been linked to SGA/IUGR.

Several studies have determined links between imprinted gene expression and aberrant fetal growth parameters, several of which were also identified in this study. Diplas and colleagues noted an association between *ZNF331* and *PEG10* abundance and fetal growth and IUGR [37], with subsequent work from the same group linking expression of other imprinted transcripts and growth [32, 38]. Strong negative correlations between *GRB10* expression in the placenta and head circumference, but not birth weight, have also been reported [11], which is consistent with our observations and mouse models suggesting that this gene is a negative regulator of fetal growth [39]. Our study also revealed that gender-specific differences in expression are frequently associated with IUGR. Such effects have previously been reported for the *PLAGL1* gene although the effect was restricted to females rather than males [24]. Consistent with the current study, the expression differences also occurred without a change in allelic methylation suggesting an involvement of transcription factor binding or underlying chromatin structure. Three of the seven genes with IUGR-associated differences were located within the *GPR1-AS1-ADAM23*-imprinted domain on chromosome 2. Imprinting of this region is regulated by the maternally methylated *GPR1-AS1* DMR, which is unique in that it regulates the acquisition of methylation on the paternal allele of the *ZDBF2* DMR [26, 27]. We observe a non-canonical imprinted histone signature for this region in placenta, an observation reported in mouse brain [27]. Targeted truncation of the *Gpr-as1*/Liz transcript by polyA cassette insertion results in concomitant decrease in *Zdbf2* abundance and postnatal growth restriction without affecting allelic methylation at *Gpr1-as* DMR [40]. This growth effect was evident towards the end of gestation and is consistent with the decreased expression that we observe throughout the domain in our IUGR samples.

One limitation of this study is the use of a single biopsy from the fetal side of the placenta. Recently, it was shown that the expression of *PHLDA2* was significantly increased at distal sampling sites compared with sites close to the umbilical cord [41]. Through the use of consistent sampling, great care was taken to ensure that the sampling sites were similar between samples. reassuringly, we did not observe great variability in methylation between multiple biopsies from the same placenta (Additional file 7) suggesting that variation would be minimal.

The recent description of hundreds of placenta-specific DMRs, which are largely restricted to the human [16], suggests that imprinting may have gained a significantly more instrumental role during evolution and careful characterization is required to dissect if they influence fetal growth. Critically, these placenta-specific maternally methylated DMRs are polymorphic in nature, a feature not observed at classic ubiquitous imprinted DMRs. Analysis of our placenta cohort from normal and complicated gestations revealed a similar frequency in polymorphic hypomethylation, implying that these regions may not influence fetal growth. However, there seems to be a subset of placenta-specific DMRs that do not show methylation variability, suggesting they are tightly regulated. Interestingly, the polymorphic nature is not only evident at the level of allelic methylation, as biallelic expression was observed at regions maintaining allelic methylation. In these rare divergent samples, our observations suggest some DMRs can adopt an unusual underlying histone signature with biallelic enrichment of H3K4 methylation. However, this histone modification is normally mutually exclusive with DNA methylation and further work is required to ensure that the observed pattern is not due to different epigenetic profiles in heterogeneous cells of the placenta.

For those regions that do present with epigenetic variability between gestations, it is currently unknown if this reflects polymorphic establishment in individual oocytes or erratic maintenance during embryonic reprogramming. To fully address these issues, single-cell experiments will need to be performed. Alternatively, this relaxation of allelic methylation may reflect the placenta’s ability to accommodate to early physiological conditions, with the variability observed as a consequence of the adaptive nature of the privileged placenta epigenome. Since genomic imprinting arises early in development, the variability we observe may serve as a biosensor for the impact of in utero environmental and maternal exposures. Environmental contaminants have been associated with subtle epigenetic changes [42]; therefore, it would be interesting to determine whether variability in placenta-specific DMR methylation changes with exposure, for example, to air pollutants or maternal smoking habits that have been shown to effect final fetal size [43–45]. However, our analysis in multiple gestation pregnancies suggests that the profile of polymorphic placenta-specific imprinting may simply indicate a random event rather than reflecting exposure.

## Conclusions

While the clinical manifestations associated with classic imprinting disorders has aided our understanding of the relevance of these genes in development, such cases are extremely rare. To date, few studies have examined the impact of subtler epigenetic variation in target tissues such as the placenta on allelic expression of imprinted transcripts in a way reminiscent of GWAS studies which have revealed the influence of genetic variation on growth [46]. With potentially hundreds of placenta-specific DMRs that could influence allelic expression, and their polymorphic nature, much work is required to understand how they impact the placenta’s key role during pregnancy. It will be imperative that future work discriminates between those placenta-specific imprinted transcripts that have little biological relevance, being a by-product of inherited methylation differences from the gametes, from those exerting an effect on pregnancy and that influence long-term adult phenotypes.

## Additional files

Additional file 1: Description of main clinical characteristic of the placenta cohort. (DOCX 18 kb)

Additional file 2: Details of the primers used for PCR amplifications in this study. (XLSX 27 kb)

Additional file 3: HM450k Infinium probe IDs and β values at known imprinted regions. The methylation β values for each of the probes within (A) ubiquitous and (B) known placenta-specific imprinted DMRs. (XLSX 1048 kb)

Additional file 4: Polymorphic STR marker analysis in placenta samples. Examples of electropherogram showing biallelic peaks and the lack of deletions or uniparental disomy in samples with isolated methylation defects at ubiquitous DMRs. (PDF 479 kb)

Additional file 5: Pyrosequencing confirmation of the aberrant placenta-specific methylation identified using HM450k methylation arrays. (A) Violin plots show the distribution of methylation for each DMR, as well as the median (white dot), mean (red line) and the interquartile range (black rectangle) are shown. Samples with hypomethylation defined by < 1.5 IQR are highlighted as green data points. The non-parametric Mann-Whitney-Wilcoxon test was used to calculate the statistical significance of the differences between IUGR and control groups (ns indicated no significance, *p* > 0.05). (B) Violin plots show the distribution of methylation for the *AIM1* and *RHOBTB3* DMR, separated according to gender (blue for male and red for female). (C) Example of bisulphite PCR and sub-cloning of samples identified as hypomethylated. Each circle represents a single CpG on a DNA strand: (•) methylated cytosine, (o) unmethylated cytosine. Each row corresponds to an individual cloned sequence with the genotype indicated for heterozygous SNP incorporated into the amplicon. (PDF 809 kb)

Additional file 6: Catalogue of outliers. Outliers identified using 1.5 IQR for placenta-specific imprinted DMRs using HM450k array dataset. (XLSX 12 kb)

Additional file 7: Analysis of placenta-specific DMRs in paired samples using the HM450k methylation arrays. (A) Heatmap of pairwise correlation coefficients of for placenta-specific DMRs in samples derived from CVS vs term placenta, multiple biopsies from the same placenta and those from multiple gestations. Numbers in the coloured squares represent the Pearson’s coefficients. (B) Heatmap of Infinium probes located in placenta-specific DMRs with loci with highly concordant methylation between samples highlighted by yellow boxes. (PDF 685 kb)

Additional file 8: Methylation profiling of the *LIN28B* DMR in dizygotic twins. Schematic representation of the *LIN28B* locus, indicating the CpG island incorporating the DMR. Characterization of allelic methylation for placenta samples PL215 and PL216 samples from a twin gestation by bisulphite PCR and sub-cloning. Each circle represents a single CpG on a DNA strand: (•) methylated cytosine, (o) unmethylated cytosine. Each row corresponds to an individual cloned sequence with the genotype indicated for heterozygous SNP incorporated into the amplicon. Quantification of total methylation at this region was performed using pyrosequencing. Gene coordinates are from hg19 genome build. (PDF 371 kb)

Additional file 9: Quantification of expression levels for imprinted transcripts in placenta samples. Microfluidic-based RT-qPCR analysis of imprinted transcripts in 50 placenta samples. Results are presented as violin plots for genes with statistically difference between IUGR and controls (Student’s two-tailed t-test, *p* < 0.05). The median (white dot), mean (red line) and the interquartile range (black rectangle) are shown. All expression levels were normalised to the mean of the *RPL19* housekeeping gene. (A) Expression difference for transcripts associated with ubiquitous DMRs. (B) Expression difference for transcripts associated with placenta-specific DMRs. (C and D) Significant expression difference between IUGR and controls separated by gender (blue for male and red for female). (PDF 582 kb)

Additional file 10: Multivariant analysis. Identification of factors/conditions that influence expression levels between IUGR and control placenta samples. (XLSX 12 kb)

## Abbreviations

ChIP: Chromatin immunoprecipitation
CVS: Chorionic villus sampling
DMRs: Differentially methylated regions
DNA: Deoxyribonucleic acid
EPIC: Illumina Infinium MethylationEPIC arrays
GWAS: Genome-wide association studies
H3K: Histone 3 lysine
HM450k: Illumina Infinium Human Methylation450 BeadChip arrays
ICR: Imprinting control regions
IQR: Interquartile range
IUGR: Intrauterine growth restriction
LOM: Loss-of-methylation
PCR: Polymerase chain reaction
PE: Pre-eclampsia
SGA: Small for gestational age
SNPs: Single nucleotide polymorphisms
SRS: Silver-Russell syndrome
