# Supplementary material for: Differences in expression rather than methylation at placenta-specific imprinted loci is associated with intrauterine growth restriction
Source: Clin Epigenetics. 2019 Feb 26;11:35. doi: 10.1186/s13148-019-0630-4 (PMC6390544; doi:10.1186/s13148-019-0630-4)
Supplement: Supplementary file 1 — Description of main clinical characteristic of the placenta cohort. (DOCX 18 kb) [file 13148_2019_630_MOESM1_ESM.docx]

| **Characteristic** | **HM450k array placenta cohort** |
| --- | --- |
| **Placenta/fetus** |  |
| # Individuals | 67 |
| Male (%) | 33 (50) |
| Gestational age at delivery*  (37> weeks)  (35-37 weeks)  (32-35 weeks)  (<32 weeks) | 48 (71)  5 (7)  7 (11)  7 (11) |
| Birth weight (g) | 2721 ± 870 |
| IUGR (%)* | 14 (21) |
| SGA (%) | 14 (21) |
| Pre-eclampsia (%) | 31 (47) |
| **Maternal** |  |
| # Individuals | 67 |
| Maternal age | 32 ± 6** |
| Parity: Primipara (%) | 39 (75) |
| Delivery: C section (%) | 25 (42) |
| Previous miscarriage | 44 (between 44 mums) |

**Cohort characteristics for samples hybridized to the Illumina HM450k array.** Categorical data are expressed as number of observations (%); continuous data are expressed as mean ± SD. Categories are not mutually exclusive. By definition, evidence of IUGR is an indication for elective premature delivery. ** data on maternal age only available for Spanish samples.

| **Characteristic** | **Non-IUGR** | **IUGR** |
| --- | --- | --- |
| **Placenta/fetus** |  |  |
| # Individuals | 76 | 51 |
| Male (%) | 35 (48) | 25 (50) |
| Multiple gestation | 28 (37) | 15 (30) |
| Gestational age at delivery*  (37> weeks)  (35-37 weeks)  (32-35 weeks)  (<32 weeks) | 32 (47)  5 (7)  21 (31)  10 (15) | 20 (45)  8 (18)  12 (28)  4 (9) |
| Birth weight (g) | 2468 ± 1015 | 1883 ± 550** |
| Placenta weight (g) | 606 ± 228 | 410 ± 149** |
| Caesarean sections (%) | 33 (45) | 32 (65)*** |
| Pre-eclampsia (%) | 2 (3) | 4 (9) |
| **Maternal** |  |  |
| # Individuals | 70 | 48 |
| Maternal age | 32.3 ± 6.0 | 32.3 ± 6.0 |
| Caucasian (%) | 50 (68) | 35 (65) |
| Parity: Primipara (%) | 50 (65) | 28 (57) |
| Delivery: C section (%) | 32 (44) | 32 (65)*** |
| Previous miscarriage | 48 (between 39 mums) | 21 (between 48 mums) |
| Smoking (self-reported information if given) | 17 (32) | 18 (43) |

**Cohort characteristics for samples analysed by pyrosequencing.** Categorical data are expressed as number of observations (%); continuous data are expressed as mean ± SD. Categories are not mutually exclusive. By definition, evidence of IUGR is an indication for elective premature delivery. ** Significantly different between non-IUGR and IUGR pregnancies (Student’s T test P < 0.05). *** Significantly different between non-IUGR and IUGR pregnancies (Chi squared P < 0.05).

Clinical definitions:

**Preeclampsia:** Pregnant women at 20 weeks’ gestation or later who have new-onset, persistent hypertension (>140mmHg systolic or > 90mmHg diastolic measurements twice at least 4hrs apart) and proteinuria.

**SGA:** A birthweight belowthe 10^th^ centile.

**Intrauterine growth restriction:** A fetus with abdominal circumference or estimated fetal weight < 10th centile and abnormal umbilical artery Doppler pulsatility index above the 95^th^percentile based on local standards. Routine ultrasounds in Spain are performed at 20 and 33 weeks of pregnancy, so diagnoses is usually at one of these time points, unless other concerns (pre-eclampsia, maternal diabetes, etc prompt additional scans).
