# Supplementary material for: Differences in expression rather than methylation at placenta-specific imprinted loci is associated with intrauterine growth restriction
Source: Clin Epigenetics. 2019 Feb 26;11:35. doi: 10.1186/s13148-019-0630-4 (PMC6390544; doi:10.1186/s13148-019-0630-4)
Supplement: Supplementary file 4 — Polymorphic STR marker analysis in placenta samples. Examples of electropherogram showing biallelic peaks and the lack of deletions or uniparental disomy in samples with isolated methylation defects at ubiquitous DMRs. (PDF 479 kb) [file 13148_2019_630_MOESM4_ESM.pdf]

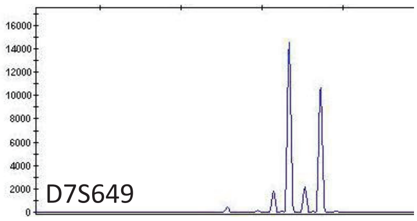

PL35 { D7S2544 ~900 kb centromeric to MEST / D7S3054 ~650 kb telomeric to MEST / D7S649 ~620 kb telomeric to MEST } Het

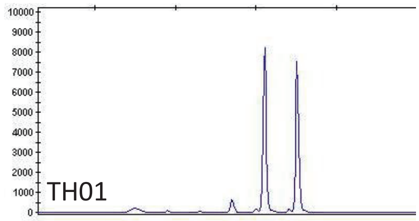

PL217 / PL67 / PL90 } TH01 ~175 kb centromeric to H19 - All HET

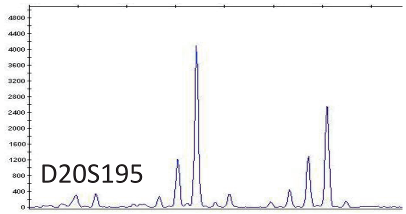

PL37 { D20S1095 ~1.7 Mb telomeric to MCTS2 / D20S174 ~7.5Mb centromeric to MCTS2 } Het
