# Supplementary figures and images for: Differences in expression rather than methylation at placenta-specific imprinted loci is associated with intrauterine growth restriction
Source: Clin Epigenetics. 2019 Feb 26;11:35. doi: 10.1186/s13148-019-0630-4 (PMC6390544; doi:10.1186/s13148-019-0630-4)

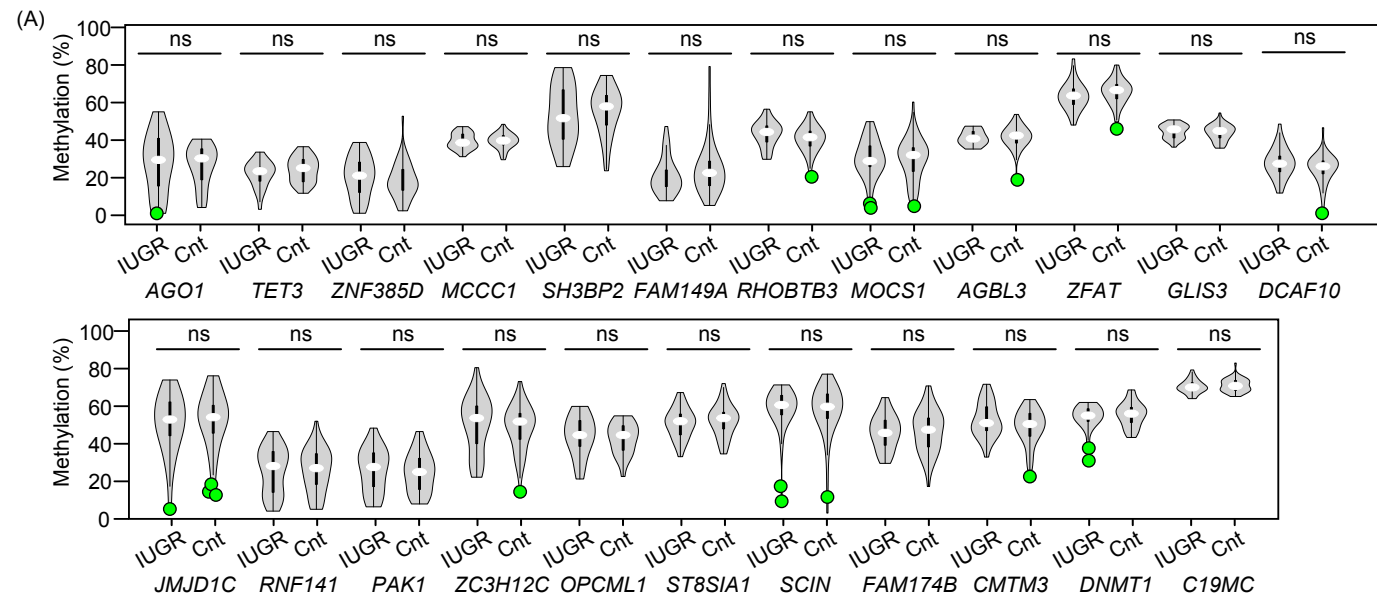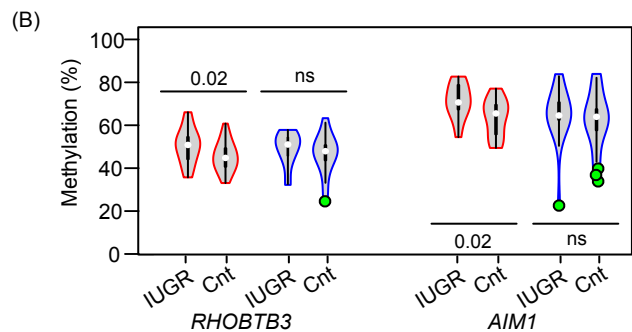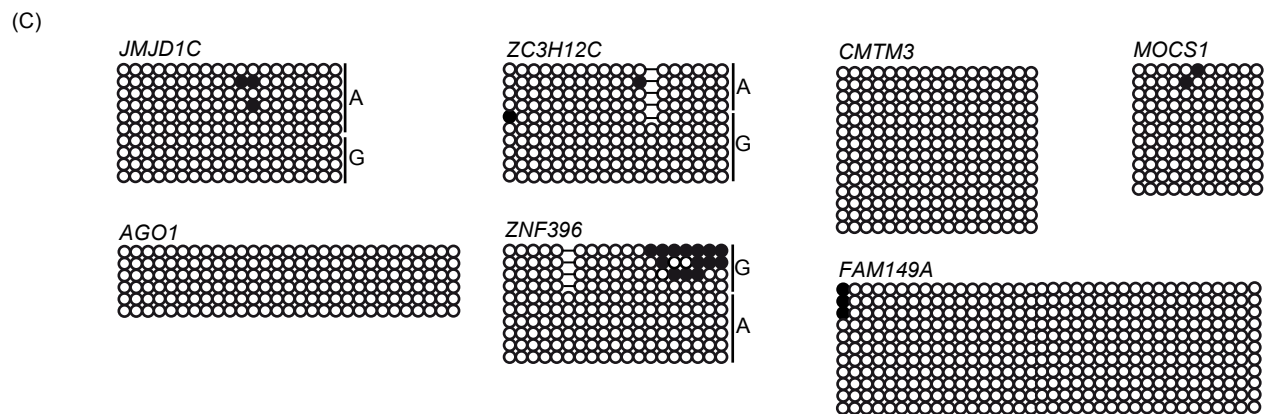

Supplement: Supplementary file 5 — Pyrosequencing confirmation of the aberrant placenta-specific methylation identified using HM450k methylation arrays. (A) Violin plots show the distribution of methylation for each DMR, as well as the median (white dot), mean (red line) and the interquartile range (black rectangle) are shown. Samples with hypomethylation defined by < 1.5 IQR are highlighted as green data points. The non-parametric Mann-Whitney-Wilcoxon test was used to calculate the statistical significance of the differences between IUGR and control groups (ns indicated no significance, p > 0.05). (B) Violin plots show the distribution of methylation for the AIM1 and RHOBTB3 DMR, separated according to gender (blue for male and red for female). (C) Example of bisulphite PCR and sub-cloning of samples identified as hypomethylated. Each circle represents a single CpG on a DNA strand: (•) methylated cytosine, (o) unmethylated cytosine. Each row corresponds to an individual cloned sequence with the genotype indicated for heterozygous SNP incorporated into the amplicon. (PDF 809 kb) [file 13148_2019_630_MOESM5_ESM.pdf]

# *LIN28B* DMR

chr6:105,400,631-105,402,559

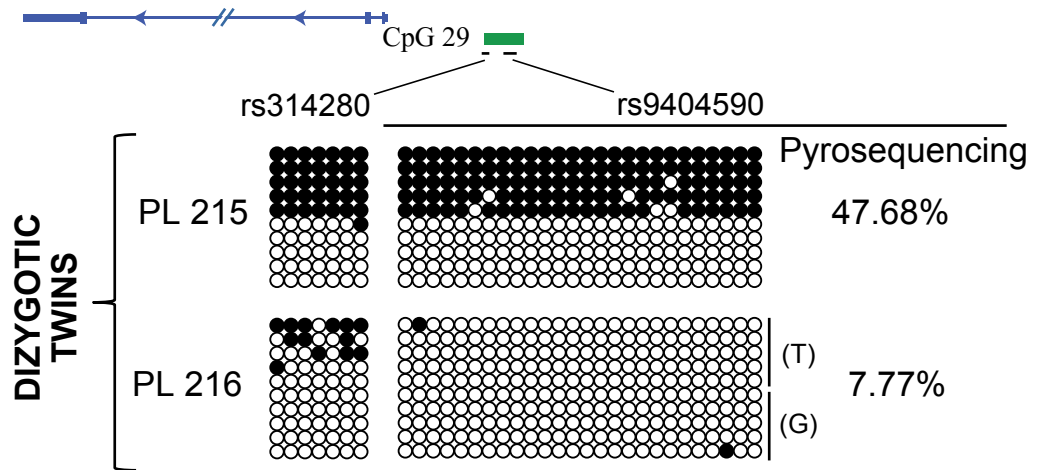

Supplement: Supplementary file 8 — Methylation profiling of the LIN28B DMR in dizygotic twins. Schematic representation of the LIN28B locus, indicating the CpG island incorporating the DMR. Characterization of allelic methylation for placenta samples PL215 and PL216 samples from a twin gestation by bisulphite PCR and sub-cloning. Each circle represents a single CpG on a DNA strand: (•) methylated cytosine, (o) unmethylated cytosine. Each row corresponds to an individual cloned sequence with the genotype indicated for heterozygous SNP incorporated into the amplicon. Quantification of total methylation at this region was performed using pyrosequencing. Gene coordinates are from hg19 genome build. (PDF 371 kb) [file 13148_2019_630_MOESM8_ESM.pdf]

(A)

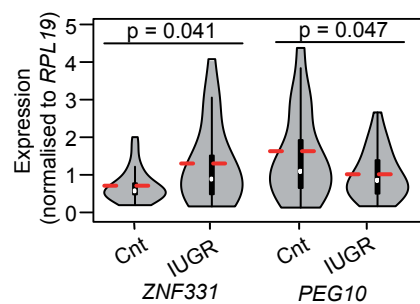

(B)

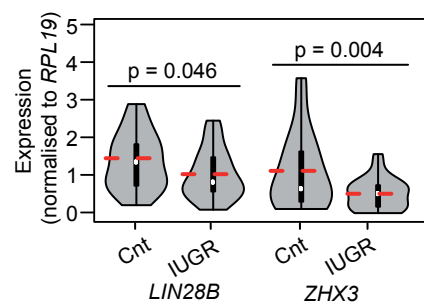

(C)

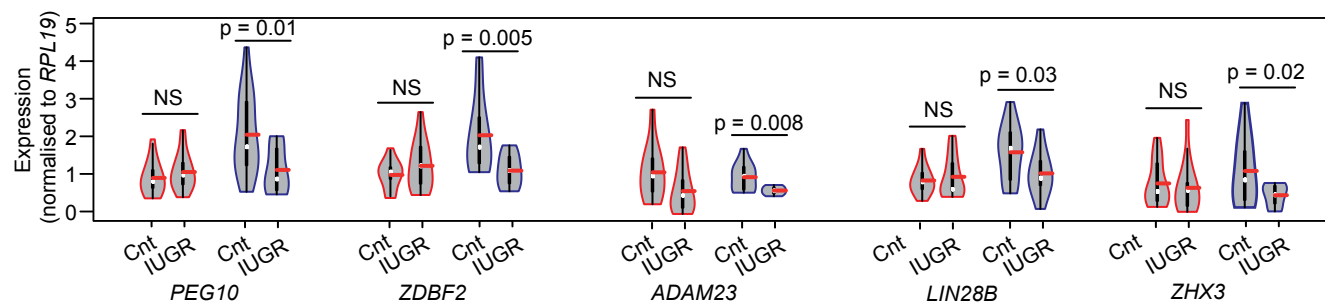

(D)

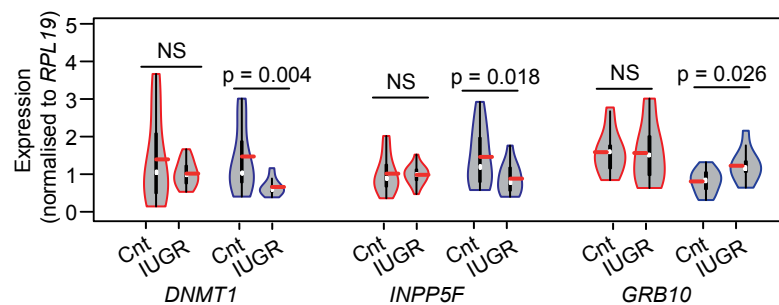

Supplement: Supplementary file 9 — Quantification of expression levels for imprinted transcripts in placenta samples. Microfluidic-based RT-qPCR analysis of imprinted transcripts in 50 placenta samples. Results are presented as violin plots for genes with statistically difference between IUGR and controls (Student’s two-tailed t-test, p < 0.05). The median (white dot), mean (red line) and the interquartile range (black rectangle) are shown. All expression levels were normalised to the mean of the RPL19 housekeeping gene. (A) Expression difference for transcripts associated with ubiquitous DMRs. (B) Expression difference for transcripts associated with placenta-specific DMRs. (C and D) Significant expression difference between IUGR and controls separated by gender (blue for male and red for female). (PDF 582 kb) [file 13148_2019_630_MOESM9_ESM.pdf]
