# Supplementary material for: Differences in expression rather than methylation at placenta-specific imprinted loci is associated with intrauterine growth restriction
Source: Clin Epigenetics. 2019 Feb 26;11:35. doi: 10.1186/s13148-019-0630-4 (PMC6390544; doi:10.1186/s13148-019-0630-4)
Supplement: Supplementary file 7 — Analysis of placenta-specific DMRs in paired samples using the HM450k methylation arrays. (A) Heatmap of pairwise correlation coefficients of for placenta-specific DMRs in samples derived from CVS vs term placenta, multiple biopsies from the same placenta and those from multiple gestations. Numbers in the coloured squares represent the Pearson’s coefficients. (B) Heatmap of Infinium probes located in placenta-specific DMRs with loci with highly concordant methylation between samples highlighted by yellow boxes. (PDF 685 kb) [file 13148_2019_630_MOESM7_ESM.pdf]

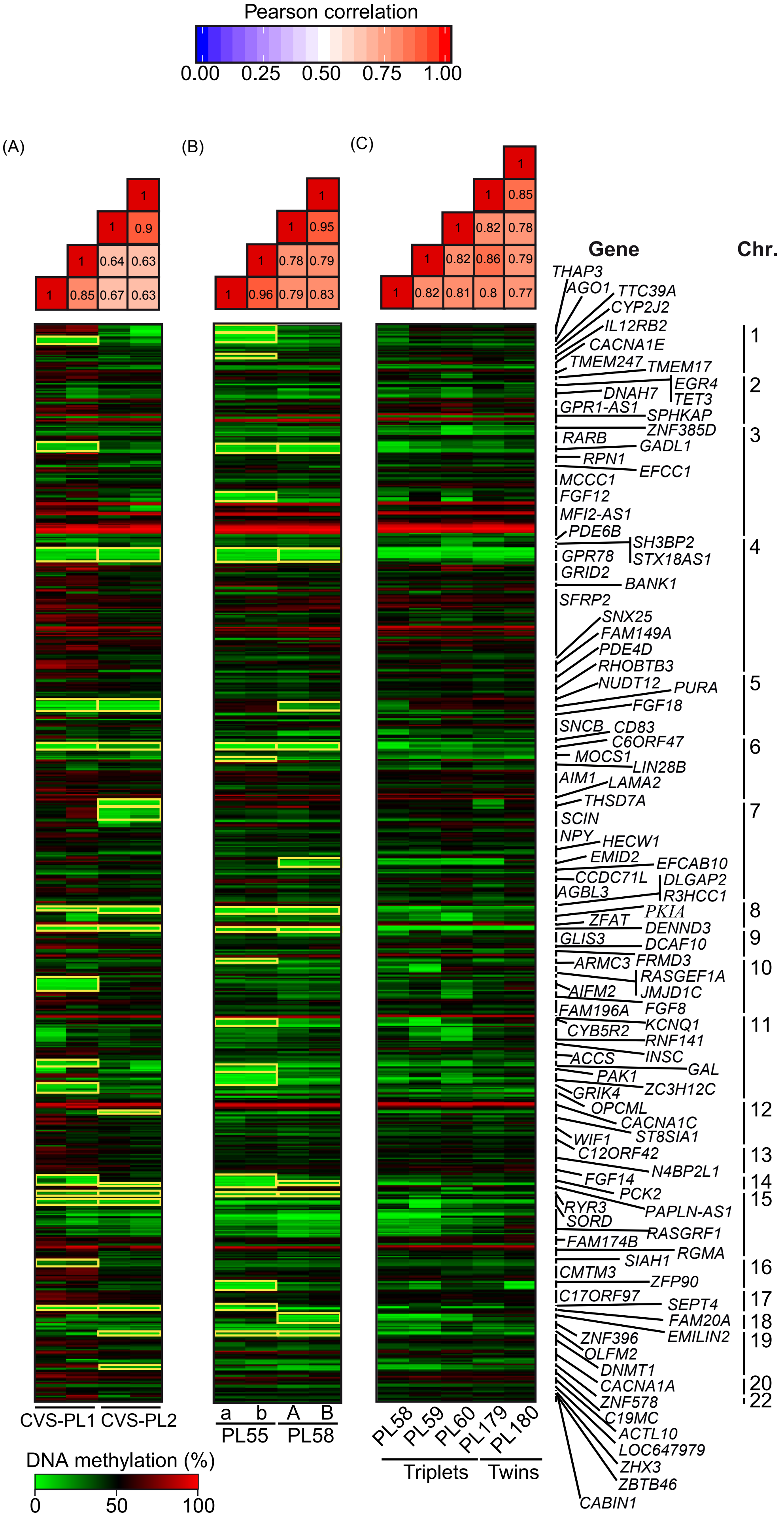

Pearson correlation
0.00 0.25 0.50 0.75 1.00
(A)
(B)
(C)
1 0.9
1 0.64 0.63
1 0.85 0.67 0.63
1 0.95
1 0.78 0.79
1 0.96 0.79 0.83
1 0.85
1 0.82 0.78
1 0.82 0.86 0.79
1 0.82 0.81 0.8 0.77
Gene
Chr.
THAP3 AGO1 TTC39A CYP2J2 IL12RB2 CACNA1E TMEM247 TMEM17 EGR4 DNAH7 TET3 GPR1-AS1 SPHKAP ZNF385D RARB GADL1 RPN1 EFCC1 MCCC1 FGF12 MFI2-AS1 PDE6B SH3BP2 GPR78 STX18AS1 GRID2 BANK1 SFRP2 SNX25 FAM149A PDE4D RHOBTB3 NUDT12 PURA FGF18 SNCB CD83 C6ORF47 MOCS1 LIN28B AIM1 LAMA2 THSD7A SCIN NPY HECW1 EMID2 EFCAB10 CCDC71L DLGAP2 AGBL3 R3HCC1 PKIA ZFAT DENND3 GLIS3 DCAF10 ARMC3 FRMD3 RASGEF1A AIFM2 JMJD1C FAM196A FGF8 CYB5R2 KCNQ1 RNF141 ACCS INSC GAL PAK1 ZC3H12C GRIK4 OPCML CACNA1C ST8SIA1 WIF1 C12ORF42 N4BP2L1 FGF14 PCK2 RYR3 PAPLN-AS1 SORD RASGRF1 FAM174B RGMA SIAH1 CMTM3 ZFP90 C17ORF97 SEPT4 FAM20A EMILIN2 ZNF396 OLFM2 DNMT1 CACNA1A ZNF578 C19MC ACTL10 LOC647979 ZHX3 ZBTB46 CABIN1
1 2 3 4 5 6 7 8 9 10 11 12 13 14 15 16 17 18 19 20 22
CVS-PL1 CVS-PL2
a b A B
PL55 PL58
PL58 PL59 PL60 PL179 PL180
Triplets Twins
DNA methylation (%)
0 50 100
